# Bis(methanol-κ*O*)bis­(1,2-diamino-2-hy­droxy­imino­ethanone oximato-κ^2^ *N*,*N*′)copper(II) bis­(oxamide dioxime) methanol disolvate

**DOI:** 10.1107/S1600536812036811

**Published:** 2012-09-01

**Authors:** Daying Liu, Ruihong Zhang, Hui Hu, Jing Qi, Guangming Yang

**Affiliations:** a Department of Chemistry, Nankai University, Tianjin 300071, People’s Republic of China

## Abstract

In the title compound, [Cu(C_2_H_5_N_4_O_2_)_2_(CH_3_OH)_2_]·2C_2_H_6_N_4_O_2_·2CH_3_OH, the Cu^II^ atom, lying on an inversion center, is coordinated by four N atoms from two 1,2-diamino-2-hy­droxy­imino­ethanone oximate anion and two O atoms from two methanol mol­ecules in a distorted octa­hedral geometry. The two uncoordinating oxamide dioxime mol­ecules, each lying on an inversion center, adopt a *trans* conformation. In the crystal, O—H⋯O, N—H⋯O and N—H⋯N hydrogen bonds link the complex mol­ecules and the oxamide dioxime and methanol mol­ecules.

## Related literature

For related structures, see: Bélombé *et al.* (2006 ▶); Belombe *et al.* (2007) ▶; Egharevba *et al.* (1982 ▶); Endres (1980 ▶); Endres & Schlicksupp (1980 ▶); Endres *et al.* (1983 ▶); Gunasekaran *et al.* (1995 ▶).
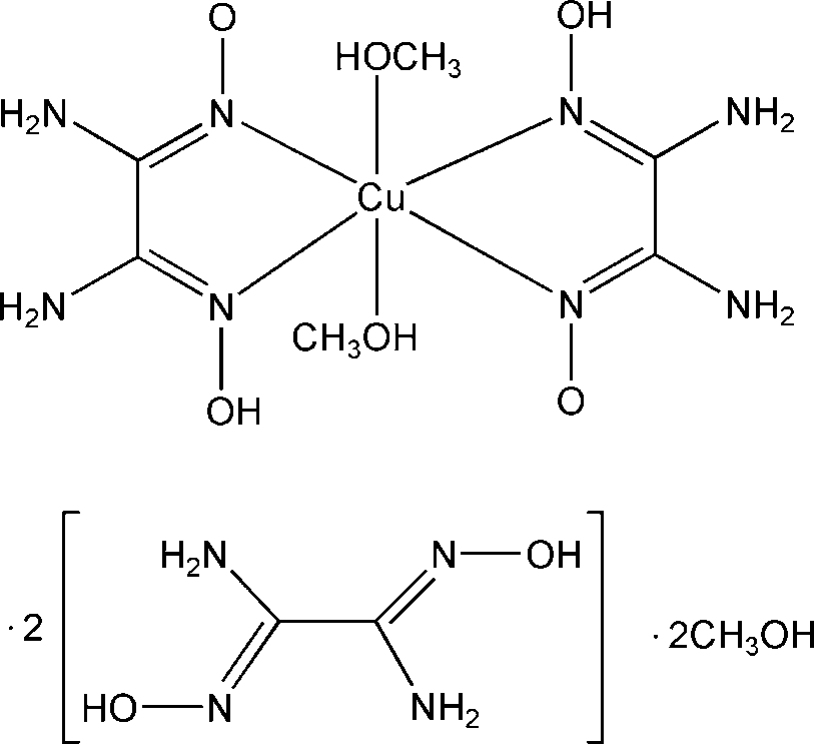


## Experimental

### 

#### Crystal data

[Cu(C_2_H_5_N_4_O_2_)_2_(CH_4_O)_2_]·2C_2_H_6_N_4_O_2_·2CH_4_O
*M* *_r_* = 662.13
Triclinic, 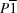

*a* = 7.567 (3) Å
*b* = 8.874 (4) Å
*c* = 10.867 (5) Å
α = 92.046 (4)°
β = 103.327 (9)°
γ = 104.957 (5)°
*V* = 682.5 (5) Å^3^
*Z* = 1
Mo *K*α radiation
μ = 0.89 mm^−1^
*T* = 113 K
0.28 × 0.24 × 0.22 mm

#### Data collection

Rigaku Saturn724 CCD diffractometer
Absorption correction: multi-scan (*CrystalClear*; Rigaku, 2009 ▶) *T*_min_ = 0.790, *T*_max_ = 0.829
7216 measured reflections
3212 independent reflections
2252 reflections with *I* > 2σ(*I*)
*R*_int_ = 0.044

#### Refinement

*R*[*F* ^2^ > 2σ(*F* ^2^)] = 0.033
*wR*(*F* ^2^) = 0.086
*S* = 0.99
3212 reflections
198 parameters
H atoms treated by a mixture of independent and constrained refinement
Δρ_max_ = 0.30 e Å^−3^
Δρ_min_ = −0.39 e Å^−3^

### 

Data collection: *CrystalClear* (Rigaku, 2009 ▶); cell refinement: *CrystalClear*; data reduction: *CrystalClear*; program(s) used to solve structure: *SHELXS97* (Sheldrick, 2008 ▶); program(s) used to refine structure: *SHELXL97* (Sheldrick, 2008 ▶); molecular graphics: *SHELXTL* (Sheldrick, 2008 ▶); software used to prepare material for publication: *SHELXTL*.

## Supplementary Material

Crystal structure: contains datablock(s) global, I. DOI: 10.1107/S1600536812036811/hy2572sup1.cif

Structure factors: contains datablock(s) I. DOI: 10.1107/S1600536812036811/hy2572Isup2.hkl

Supplementary material file. DOI: 10.1107/S1600536812036811/hy2572Isup4.cdx

Additional supplementary materials:  crystallographic information; 3D view; checkCIF report

## Figures and Tables

**Table 1:** Hydrogen-bond geometry (Å, °)

*D*—H⋯*A*	*D*—H	H⋯*A*	*D*⋯*A*	*D*—H⋯*A*
O2—H2⋯O1^i^	0.84	1.96	2.737 (3)	154
O3—H3⋯O5^ii^	0.84	1.92	2.744 (3)	166
O4—H4⋯O1^iii^	0.84	1.79	2.621 (3)	169
O5—H5⋯O1^iv^	0.81 (2)	1.87 (3)	2.657 (3)	164 (2)
O6—H6⋯O5^v^	0.79 (3)	1.94 (3)	2.721 (3)	173 (3)
N3—H3*A*⋯N7^vi^	0.88	2.50	3.195 (3)	136
N3—H3*B*⋯O6^vii^	0.88	2.32	3.167 (3)	162
N4—H4*A*⋯O4^viii^	0.88	2.35	3.112 (3)	145
N4—H4*B*⋯O6^vii^	0.88	2.00	2.878 (3)	172
N6—H6*A*⋯O3^ix^	0.88	2.26	3.097 (3)	159
N6—H6*B*⋯N7^vii^	0.88	2.19	3.007 (3)	155
N8—H8*A*⋯O4^ix^	0.88	2.20	3.043 (3)	159
N8—H8*B*⋯N5^x^	0.88	2.21	3.031 (3)	154

Symmetry codes: (i) 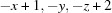; (ii) 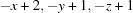; (iii) 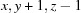; (iv) 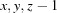; (v) 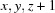; (vi) 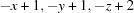; (vii) 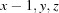; (viii) 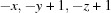; (ix) 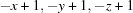; (x) 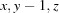.

**Fig. 1.**
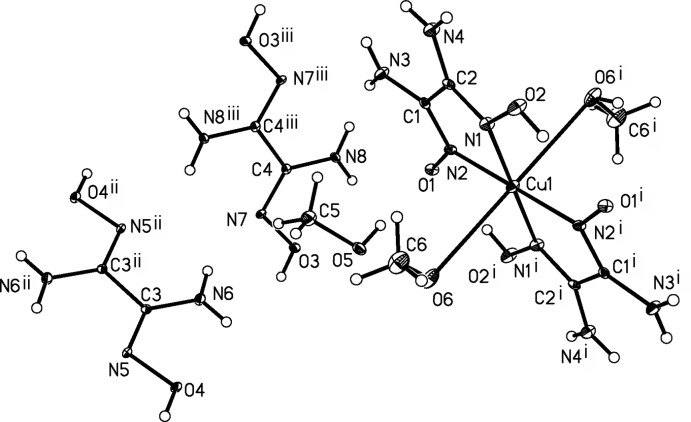
The molecular structure of the title compound. Displacement ellipsoids are drawn at the 30% probability level. [Symmetry codes: (i) 1-x, -y, 2-z; (ii) -x, 2-y, 1-z; (iii) 1-x, 1-y, 1-z.]

## supplementary crystallographic information

### Comment

Owing to the variety of structures and unique properties, transition metal complexes of oxamide oxime (diaminoglyoxime, oaoH_2_) are of great interest. So far, most of the published work concerns 4-coordinated transition metal oxamide oximate complexs (Endres, 1980; Endres & Schlicksupp, 1980; Endres *et al.*, 1983). The 6-coordinated transition metal oxamide oximate complexes have not been reported hitherto (Bélombé *et al.*, 2006; Belombe *et al.*, 2007; Egharevba *et al.*, 1982). We used oxamide oxime as ligands (Gunasekaran *et al.*, 1995) and obtained green crystals of the title compound from a methanol solution.

In the title compound, the Cu^II^ atom, lying on an inversion center, is surrounded in an octahedral environment defined by four N atoms from two oaoH ligands and two O atoms from two methanol molecules (Fig. 1). The methanol molecules are weakly coordinated to the Cu atom with a Cu—O distance of 2.797 (2) Å. In the crystal, O—H···O, N—H···O and N—H···N hydrogen bonds (Table 1) link the complex molecules and the oxamide oxime and methanol molecules.

### Experimental

A methanol solution (10 ml) of copper acetate (0.1 mmol) was added dropwise to a methanol solution (10 ml) of oxamide oxime (0.1 mmol). The title compound was obtained as green crystals by slow evaporation of the filtrate in air at room temperature 5 days later. Analysis, calculated for C_12_H_38_CuN_16_O_12_: C 21.77, H 5.78, N 33.85, O 29.00%; found: C 21.79, H 5.76, N 33.88, O 29.02%.

### Refinement

H atoms of methanol molecules were located from a difference Fourier map and refined isotropically with *U*_iso_(H) = 1.5*U*_eq_(O). The other H atoms were positioned geometrically and refined as riding atoms, with C—H = 0.98, N—H = 0.88 and O—H = 0.84 Å and with *U*_iso_(H) = 1.2(1.5 for methyl and hydroxyl)*U*_eq_(C,N,O).

### Crystal data

**Table:**

[Cu(C_2_H_5_N_4_O_2_)_2_(CH_4_O)_2_]·2C_2_H_6_N_4_O_2_·2CH_4_O	*Z* = 1
*M**_r_* = 662.13	*F*(000) = 347
Triclinic, *P*1	*D*_x_ = 1.611 Mg m^−^^3^
Hall symbol: -P 1	Mo *K*α radiation, λ = 0.71073 Å
*a* = 7.567 (3) Å	Cell parameters from 2266 reflections
*b* = 8.874 (4) Å	θ = 1.9–27.9°
*c* = 10.867 (5) Å	µ = 0.89 mm^−^^1^
α = 92.046 (4)°	*T* = 113 K
β = 103.327 (9)°	Block, green
γ = 104.957 (5)°	0.28 × 0.24 × 0.22 mm
*V* = 682.5 (5) Å^3^	

### Data collection

**Table:**

Rigaku Saturn724 CCD diffractometer	3212 independent reflections
Radiation source: rotating anode	2252 reflections with *I* > 2σ(*I*)
Multilayer monochromator	*R*_int_ = 0.044
Detector resolution: 14.22 pixels mm^-1^	θ_max_ = 27.9°, θ_min_ = 1.9°
ω scans	*h* = −9→9
Absorption correction: multi-scan (*CrystalClear*; Rigaku, 2009)	*k* = −11→11
*T*_min_ = 0.790, *T*_max_ = 0.829	*l* = −11→14
7216 measured reflections	

### Refinement

**Table:**

Refinement on *F*^2^	Primary atom site location: structure-invariant direct methods
Least-squares matrix: full	Secondary atom site location: difference Fourier map
*R*[*F*^2^ > 2σ(*F*^2^)] = 0.033	Hydrogen site location: inferred from neighbouring sites
*wR*(*F*^2^) = 0.086	H atoms treated by a mixture of independent and constrained refinement
*S* = 0.99	*w* = 1/[σ^2^(*F*_o_^2^) + (0.0231*P*)^2^] where *P* = (*F*_o_^2^ + 2*F*_c_^2^)/3
3212 reflections	(Δ/σ)_max_ = 0.001
198 parameters	Δρ_max_ = 0.30 e Å^−^^3^
0 restraints	Δρ_min_ = −0.39 e Å^−^^3^

### Special details

**Table:**

Geometry. All e.s.d.'s (except the e.s.d. in the dihedral angle between two l.s. planes) are estimated using the full covariance matrix. The cell e.s.d.'s are taken into account individually in the estimation of e.s.d.'s in distances, angles and torsion angles; correlations between e.s.d.'s in cell parameters are only used when they are defined by crystal symmetry. An approximate (isotropic) treatment of cell e.s.d.'s is used for estimating e.s.d.'s involving l.s. planes.
Refinement. Refinement of *F*^2^ against ALL reflections. The weighted *R*-factor *wR* and goodness of fit *S* are based on *F*^2^, conventional *R*-factors *R* are based on *F*, with *F* set to zero for negative *F*^2^. The threshold expression of *F*^2^ > σ(*F*^2^) is used only for calculating *R*-factors(gt) *etc*. and is not relevant to the choice of reflections for refinement. *R*-factors based on *F*^2^ are statistically about twice as large as those based on *F*, and *R*- factors based on ALL data will be even larger.

### Fractional atomic coordinates and isotropic or equivalent isotropic displacement parameters (Å^2^)

**Table:**

	*x*	*y*	*z*	*U*_iso_*/*U*_eq_	
C1	0.2733 (3)	0.1538 (3)	1.0896 (2)	0.0102 (5)	
C2	0.2003 (3)	0.1293 (3)	0.9482 (2)	0.0102 (5)	
C3	0.0546 (3)	0.9542 (3)	0.47271 (19)	0.0089 (5)	
C4	0.5721 (3)	0.4615 (3)	0.53280 (19)	0.0092 (5)	
C5	0.6807 (3)	0.4989 (3)	0.2349 (2)	0.0207 (6)	
H5A	0.7195	0.5685	0.3141	0.031*	
H5B	0.5433	0.4548	0.2126	0.031*	
H5C	0.7183	0.5586	0.1666	0.031*	
C6	0.7357 (4)	0.3711 (3)	0.9294 (2)	0.0317 (7)	
H6C	0.7999	0.4807	0.9621	0.048*	
H6D	0.5989	0.3535	0.9151	0.048*	
H6E	0.7658	0.3478	0.8490	0.048*	
Cu1	0.5000	0.0000	1.0000	0.01296 (13)	
N1	0.3058 (2)	0.0743 (2)	0.89207 (16)	0.0131 (4)	
N2	0.4170 (2)	0.0981 (2)	1.12976 (16)	0.0105 (4)	
N3	0.1956 (2)	0.2296 (2)	1.16254 (18)	0.0165 (5)	
H3A	0.2423	0.2452	1.2454	0.020*	
H3B	0.0982	0.2636	1.1274	0.020*	
N4	0.0410 (2)	0.1635 (2)	0.89006 (17)	0.0163 (5)	
H4A	0.0011	0.1485	0.8067	0.020*	
H4B	−0.0236	0.2010	0.9353	0.020*	
N5	0.1797 (2)	1.0348 (2)	0.41876 (16)	0.0100 (4)	
N6	0.0166 (3)	0.7998 (2)	0.48310 (18)	0.0168 (5)	
H6A	0.0792	0.7436	0.4517	0.020*	
H6B	−0.0710	0.7546	0.5214	0.020*	
N7	0.7401 (2)	0.5552 (2)	0.58021 (17)	0.0118 (4)	
N8	0.5201 (2)	0.3067 (2)	0.53953 (18)	0.0172 (5)	
H8A	0.6030	0.2596	0.5788	0.021*	
H8B	0.4030	0.2519	0.5047	0.021*	
O1	0.50028 (19)	0.11732 (19)	1.25853 (13)	0.0127 (4)	
O2	0.2404 (2)	0.0421 (2)	0.76012 (14)	0.0200 (4)	
H2	0.3155	0.0041	0.7315	0.030*	
O3	0.86131 (19)	0.46482 (19)	0.63897 (15)	0.0158 (4)	
H3	0.9731	0.5217	0.6610	0.024*	
O4	0.2651 (2)	0.93020 (19)	0.36628 (14)	0.0130 (4)	
H4	0.3493	0.9817	0.3336	0.019*	
O5	0.7699 (2)	0.3745 (2)	0.25156 (15)	0.0168 (4)	
H5	0.688 (3)	0.305 (3)	0.267 (2)	0.025*	
O6	0.7980 (2)	0.2702 (2)	1.02027 (16)	0.0238 (5)	
H6	0.791 (4)	0.308 (3)	1.085 (3)	0.036*	

### Atomic displacement parameters (Å^2^)

**Table:**

	*U*^11^	*U*^22^	*U*^33^	*U*^12^	*U*^13^	*U*^23^
C1	0.0102 (11)	0.0089 (13)	0.0107 (12)	−0.0007 (10)	0.0048 (9)	0.0005 (9)
C2	0.0085 (11)	0.0089 (13)	0.0121 (12)	−0.0014 (10)	0.0041 (9)	0.0038 (9)
C3	0.0072 (11)	0.0099 (13)	0.0082 (11)	0.0025 (10)	−0.0006 (9)	0.0002 (9)
C4	0.0116 (11)	0.0100 (13)	0.0073 (11)	0.0026 (10)	0.0054 (9)	0.0003 (9)
C5	0.0196 (13)	0.0204 (16)	0.0233 (14)	0.0076 (12)	0.0047 (11)	0.0038 (11)
C6	0.0400 (17)	0.0313 (19)	0.0320 (17)	0.0188 (15)	0.0132 (13)	0.0151 (14)
Cu1	0.0131 (2)	0.0178 (3)	0.0095 (2)	0.00752 (19)	0.00211 (16)	0.00014 (17)
N1	0.0129 (10)	0.0201 (13)	0.0062 (10)	0.0060 (9)	0.0005 (8)	0.0006 (8)
N2	0.0082 (9)	0.0146 (11)	0.0079 (10)	0.0026 (8)	0.0010 (7)	0.0004 (8)
N3	0.0149 (10)	0.0232 (13)	0.0136 (11)	0.0109 (10)	0.0019 (8)	−0.0016 (9)
N4	0.0150 (10)	0.0236 (13)	0.0110 (10)	0.0086 (10)	0.0013 (8)	−0.0006 (9)
N5	0.0102 (9)	0.0090 (11)	0.0126 (10)	0.0047 (8)	0.0038 (8)	0.0002 (8)
N6	0.0185 (11)	0.0108 (12)	0.0279 (12)	0.0046 (9)	0.0177 (9)	0.0049 (9)
N7	0.0083 (9)	0.0109 (11)	0.0171 (10)	0.0055 (8)	0.0017 (8)	0.0025 (8)
N8	0.0085 (9)	0.0082 (11)	0.0307 (12)	0.0021 (9)	−0.0032 (8)	0.0016 (9)
O1	0.0122 (8)	0.0186 (10)	0.0067 (8)	0.0030 (7)	0.0025 (6)	0.0010 (7)
O2	0.0206 (9)	0.0349 (12)	0.0080 (8)	0.0158 (9)	0.0013 (7)	−0.0009 (8)
O3	0.0085 (8)	0.0121 (10)	0.0238 (9)	0.0039 (7)	−0.0030 (7)	0.0029 (7)
O4	0.0143 (8)	0.0118 (9)	0.0185 (9)	0.0053 (7)	0.0131 (7)	0.0032 (7)
O5	0.0125 (9)	0.0142 (11)	0.0210 (10)	−0.0002 (8)	0.0031 (7)	0.0030 (8)
O6	0.0330 (10)	0.0288 (12)	0.0191 (10)	0.0202 (9)	0.0114 (8)	0.0069 (8)

### Geometric parameters (Å, º)

**Table:**

C1—N2	1.300 (3)	Cu1—N2^iii^	1.9349 (18)
C1—N3	1.344 (3)	Cu1—N2	1.9349 (18)
C1—C2	1.496 (3)	Cu1—O6	2.797 (2)
C2—N1	1.285 (3)	N1—O2	1.397 (2)
C2—N4	1.340 (3)	N2—O1	1.380 (2)
C3—N5	1.299 (3)	N3—H3A	0.8800
C3—N6	1.340 (3)	N3—H3B	0.8800
C3—C3^i^	1.494 (4)	N4—H4A	0.8800
C4—N7	1.302 (3)	N4—H4B	0.8800
C4—N8	1.337 (3)	N5—O4	1.430 (2)
C4—C4^ii^	1.493 (4)	N6—H6A	0.8800
C5—O5	1.431 (3)	N6—H6B	0.8800
C5—H5A	0.9800	N7—O3	1.428 (2)
C5—H5B	0.9800	N8—H8A	0.8800
C5—H5C	0.9800	N8—H8B	0.8800
C6—O6	1.437 (3)	O2—H2	0.8400
C6—H6C	0.9800	O3—H3	0.8400
C6—H6D	0.9800	O4—H4	0.8400
C6—H6E	0.9800	O5—H5	0.81 (3)
Cu1—N1^iii^	1.9314 (18)	O6—H6	0.79 (2)
Cu1—N1	1.9314 (18)		
			
N2—C1—N3	125.9 (2)	N1—Cu1—O6	97.31 (8)
N2—C1—C2	112.90 (18)	N2^iii^—Cu1—O6	90.37 (7)
N3—C1—C2	121.17 (19)	N2—Cu1—O6	89.63 (7)
N1—C2—N4	125.4 (2)	C2—N1—O2	115.67 (16)
N1—C2—C1	112.98 (18)	C2—N1—Cu1	116.25 (15)
N4—C2—C1	121.63 (19)	O2—N1—Cu1	126.33 (13)
N5—C3—N6	126.10 (19)	C1—N2—O1	118.35 (17)
N5—C3—C3^i^	115.4 (3)	C1—N2—Cu1	115.95 (15)
N6—C3—C3^i^	118.5 (3)	O1—N2—Cu1	125.67 (13)
N7—C4—N8	125.9 (2)	C1—N3—H3A	120.0
N7—C4—C4^ii^	115.2 (3)	C1—N3—H3B	120.0
N8—C4—C4^ii^	118.8 (2)	H3A—N3—H3B	120.0
O5—C5—H5A	109.5	C2—N4—H4A	120.0
O5—C5—H5B	109.5	C2—N4—H4B	120.0
H5A—C5—H5B	109.5	H4A—N4—H4B	120.0
O5—C5—H5C	109.5	C3—N5—O4	108.74 (18)
H5A—C5—H5C	109.5	C3—N6—H6A	120.0
H5B—C5—H5C	109.5	C3—N6—H6B	120.0
O6—C6—H6C	109.5	H6A—N6—H6B	120.0
O6—C6—H6D	109.5	C4—N7—O3	108.65 (19)
H6C—C6—H6D	109.5	C4—N8—H8A	120.0
O6—C6—H6E	109.5	C4—N8—H8B	120.0
H6C—C6—H6E	109.5	H8A—N8—H8B	120.0
H6D—C6—H6E	109.5	N1—O2—H2	109.5
N1^iii^—Cu1—N1	179.999 (1)	N7—O3—H3	109.5
N1^iii^—Cu1—N2^iii^	80.90 (8)	N5—O4—H4	109.5
N1—Cu1—N2^iii^	99.10 (8)	C5—O5—H5	101.6 (18)
N1^iii^—Cu1—N2	99.10 (8)	C6—O6—Cu1	108.13 (15)
N1—Cu1—N2	80.90 (8)	C6—O6—H6	104 (2)
N2^iii^—Cu1—N2	179.999 (1)	Cu1—O6—H6	97 (2)
N1^iii^—Cu1—O6	82.69 (8)		
			
N2—C1—C2—N1	−7.5 (3)	N3—C1—N2—Cu1	−178.33 (18)
N3—C1—C2—N1	171.1 (2)	C2—C1—N2—Cu1	0.2 (3)
N2—C1—C2—N4	172.8 (2)	N1^iii^—Cu1—N2—C1	−175.60 (18)
N3—C1—C2—N4	−8.6 (4)	N1—Cu1—N2—C1	4.40 (18)
N4—C2—N1—O2	−3.1 (4)	O6—Cu1—N2—C1	101.86 (18)
C1—C2—N1—O2	177.20 (19)	N1^iii^—Cu1—N2—O1	6.43 (18)
N4—C2—N1—Cu1	−168.98 (19)	N1—Cu1—N2—O1	−173.57 (18)
C1—C2—N1—Cu1	11.3 (3)	O6—Cu1—N2—O1	−76.10 (17)
N2^iii^—Cu1—N1—C2	171.00 (17)	N6—C3—N5—O4	2.8 (3)
N2—Cu1—N1—C2	−9.00 (17)	C3^i^—C3—N5—O4	−177.3 (2)
O6—Cu1—N1—C2	−97.45 (18)	N8—C4—N7—O3	1.0 (3)
N2^iii^—Cu1—N1—O2	6.80 (19)	C4^ii^—C4—N7—O3	−179.8 (2)
N2—Cu1—N1—O2	−173.20 (19)	N1^iii^—Cu1—O6—C6	169.14 (15)
O6—Cu1—N1—O2	98.35 (17)	N1—Cu1—O6—C6	−10.87 (15)
N3—C1—N2—O1	−0.2 (4)	N2^iii^—Cu1—O6—C6	88.36 (15)
C2—C1—N2—O1	178.31 (17)	N2—Cu1—O6—C6	−91.64 (15)

Symmetry codes: (i) −*x*, −*y*+2, −*z*+1; (ii) −*x*+1, −*y*+1, −*z*+1; (iii) −*x*+1, −*y*, −*z*+2.

### Hydrogen-bond geometry (Å, º)

**Table:**

*D*—H···*A*	*D*—H	H···*A*	*D*···*A*	*D*—H···*A*
O2—H2···O1^iii^	0.84	1.96	2.737 (3)	154
O3—H3···O5^iv^	0.84	1.92	2.744 (3)	166
O4—H4···O1^v^	0.84	1.79	2.621 (3)	169
O5—H5···O1^vi^	0.81 (2)	1.87 (3)	2.657 (3)	164 (2)
O6—H6···O5^vii^	0.79 (3)	1.94 (3)	2.721 (3)	173 (3)
N3—H3*A*···N7^viii^	0.88	2.50	3.195 (3)	136
N3—H3*B*···O6^ix^	0.88	2.32	3.167 (3)	162
N4—H4*A*···O4^x^	0.88	2.35	3.112 (3)	145
N4—H4*B*···O6^ix^	0.88	2.00	2.878 (3)	172
N6—H6*A*···O3^ii^	0.88	2.26	3.097 (3)	159
N6—H6*B*···N7^ix^	0.88	2.19	3.007 (3)	155
N8—H8*A*···O4^ii^	0.88	2.20	3.043 (3)	159
N8—H8*B*···N5^xi^	0.88	2.21	3.031 (3)	154

Symmetry codes: (ii) −*x*+1, −*y*+1, −*z*+1; (iii) −*x*+1, −*y*, −*z*+2; (iv) −*x*+2, −*y*+1, −*z*+1; (v) *x*, *y*+1, *z*−1; (vi) *x*, *y*, *z*−1; (vii) *x*, *y*, *z*+1; (viii) −*x*+1, −*y*+1, −*z*+2; (ix) *x*−1, *y*, *z*; (x) −*x*, −*y*+1, −*z*+1; (xi) *x*, *y*−1, *z*.
